# Chemical Cross-Linking of Corneal Tissue to Reduce Progression of Loss of Sight in Patients With Keratoconus

**DOI:** 10.1167/tvst.10.5.6

**Published:** 2021-04-29

**Authors:** Atikah Haneef, Ramprasad Obula Giridhara Gopalan, Divya T. Rajendran, Jessica Nunes, Dharmalingam Kuppamuthu, Naveen Radhakrishnan, Tai-Horng Young, Hao-Ying Hsieh, Namperumalsamy Venkatesh Prajna, Colin E. Willoughby, Rachel Williams

**Affiliations:** 1 Department of Eye and Vision Science, Institute of Life Course and Medical Sciences, University of Liverpool, Liverpool, UK; 2 Aravind Medical Research Foundation, Madurai, Tamil Nadu, India; 3 Cornea Clinic, Aravind Eye Hospital, Madurai, Tamil Nadu, India; 4 Department of Biomedical Engineering, National Taiwan University, Taipei, Taiwan

**Keywords:** cross-linking, cornea, collagen, keratoconus, carbodi-imide chemistry

## Abstract

**Purpose:**

We aimed to develop a novel chemical cross-linker treatment for keratoconus by reacting dicarboxylic acid spacer molecules and amine functional groups on protein structure of the tissue using carbodi-imide chemistry. We propose this as an alternative to conventional cross-linking treatment for keratoconus.

**Methods:**

The study involved optimization of the cross-linker formulation. Mechanical stiffness of ex vivo porcine and human corneas after application of the cross-linker was measured. Histochemical analysis was performed to record changes in gross morphology after cross-linker treatment on ex vivo porcine and human and in vivo rabbit corneas. Terminal deoxynucleotidyl transferase–mediated dUTP-X nick-end-labeling (TUNEL) staining was performed to study apoptotic effects of cross-linker. Cytotoxicity potential of cross-linker was evaluated by studying explant cultures for cellular outgrowth and immunostaining assays on porcine and human corneas after treatment.

**Results:**

We demonstrated a clinically relevant increase in stiffness in ex vivo experiments using porcine and human cornea without removal of corneal epithelium. Histological analysis showed no change in gross morphology of cornea and no evidence of apoptosis. In vivo treatment of rabbit eyes demonstrated initial thinning of corneal epithelium that recovered after seven days although with abnormal regularity of cells. Cellular outgrowth from corneal explant cultures after treatment further confirmed cell survival after treatment.

**Conclusions:**

This chemical cross-linking of corneal tissue has potential advantages over current therapeutic options including lower cytotoxicity to stromal cells than ultraviolet A treatment.

**Translational Relevance:**

The cross-linker has potential to become a treatment for keratoconus because it overcomes the need for procedures using specialized equipment and ensures accessibility to large populations.

## Introduction

Keratoconus is a progressive corneal disease, resulting in thinning and ectasia of the cornea with resultant visual impairment.^1^ Although the underlying causes of keratoconus are not fully understood, there is evidence of a disruption of collagen fibrils, resulting in a change in shape and thinning of the cornea^2^; it is proposed this results from alterations in the corneal collagen lamellae because of the loss of attachments between the collagen fibrils and the proteoglycan matrix.^3^ Photochemical corneal collagen cross-linking is an FDA approved therapy to treat keratoconus.^2^

Conventional cross-linking is achieved by application of riboflavin onto the cornea after removal of the corneal epithelium, such that it penetrates the corneal stroma, followed by activation with ultraviolet A (UVA) irradiation. Riboflavin absorbs the UVA energy and is activated to its triplet state where photopolymerization results in covalent bonds between collagen molecules or with proteoglycans within the tissue. Exact macromolecular alterations in corneal cross-linking with riboflavin are not fully understood, but many ex vivo and in vivo studies have demonstrated significant and clinically relevant increases in the mechanical properties of the cornea.^4^^,^^5^ Histologically there is an increase in the collagen fiber diameter, and biochemically there is evidence of some increase in intrafibrillary collagen cross-links after riboflavin/UVA corneal cross-linking.^6^ The effectiveness of corneal cross-linking relies on the ability of the riboflavin to penetrate into the corneal stroma, for there to be sufficient oxygen present in the tissue and for the UVA to have sufficient energy to activate the reactive oxygen species. These prerequisites result in several limitations and clinical risks. The UVA required is cytotoxic, and it can take up to 12 months for keratocytes to repopulate the corneal stroma.^7^^,^^8^ UVA radiation will irreparably damage the corneal endothelium, and so, patients with a cornea thinner than 400 µm cannot be treated by this procedure.^9^^,^^10^ To achieve penetration of the riboflavin requires removal of the corneal epithelium, which is painful for the patient for several days after treatment and increases risk of infection.^11^^–^^13^ Transepithelial protocols have been evaluated but are not as effective.^4^

We hypothesized that the use of a chemical cross-linker would increase the mechanical properties of the cornea while reducing or removing the limitations and clinical risks associated with conventional corneal cross-linking in keratoconus. Chemical cross-linking would not require UVA exposure to form chemical bonds between the proteins in the stroma. We have used the well-known peptide cross-linking agents 1-ethyl-3-(3-dimethylaminopropyl) carbodi-imide HCl (EDCI) and N-hydroxysuccinimide (NHS) which activate carboxylate groups to react with primary amine residues on amino acids by forming either O-acylisourea or N-hydroxysuccinimidyl-ester intermediates as a mechanism to strengthen the corneal structure.^14^ We have included an eight carbon chain dicarboxylic acid, octanedioic acid (ODA), as a cross-linking spacer unit to provide a degree of control over corneal stiffening and prevent the tissue becoming too brittle.^15^ We demonstrated equivalent stiffening of ex vivo porcine and human cornea to the conventional UVA/riboflavin corneal cross-linking without requiring removal of the corneal epithelium and with a lower cytotoxicity in the cellular population of the cornea compared with UVA treatment, including the delicate and clinically vital corneal endothelium. In a preliminary short-term in vivo study, the treatment caused thinning of the epithelium at early time points and recovery of the epithelial layer by day seven, although with some impact on the regular thickness and arrangement of the epithelial layer. No changes in other tissue layers were observed histologically, and no apoptosis of cells was observed throughout the cornea using TUNEL staining. Chemical cross-linking of corneal tissue has potential to become a treatment for keratoconus with significant advantages over current therapeutic options.

## Methods

### Preparation of Cross-Linker Solution

All chemicals were from Sigma-Aldrich Corp. (St. Louis, MO, USA), unless otherwise stated. The cross-linker solution used was prepared as a mixture of EDCI, NHS, and ODA. NHS (0.319 g) was dissolved in 3.5 mL phosphate buffered saline solution (PBS), and EDCI (0.537 g) was dissolved in 4.0 mL PBS. NHS and EDCI are water soluble and dissolve after some mixing to give clear, solutions. ODA (0.483 g) was dissolved in 7.5 mL PBS with the addition of 2-(dimethylamino) ethanol (500 µL) and vigorous mixing to give a clear solution. The three solutions were added together and vortexed, giving a 0.2 M solution with a 1:1:1 molar ratio of the three components. The stock solution was further diluted with PBS to give a 0.02 M solution. PBS or triton-X solution (16.7%, 5:30 v/v in PBS) instead of the cross-linking solution was used as controls. Hexanedioic acid (HDA) and decanedioic acid (DDA) were kindly provided ready dissolved by SpheriTech Ltd., Runcorn, UK.

### Treating the Tissue

#### Porcine

**Figure 1.**
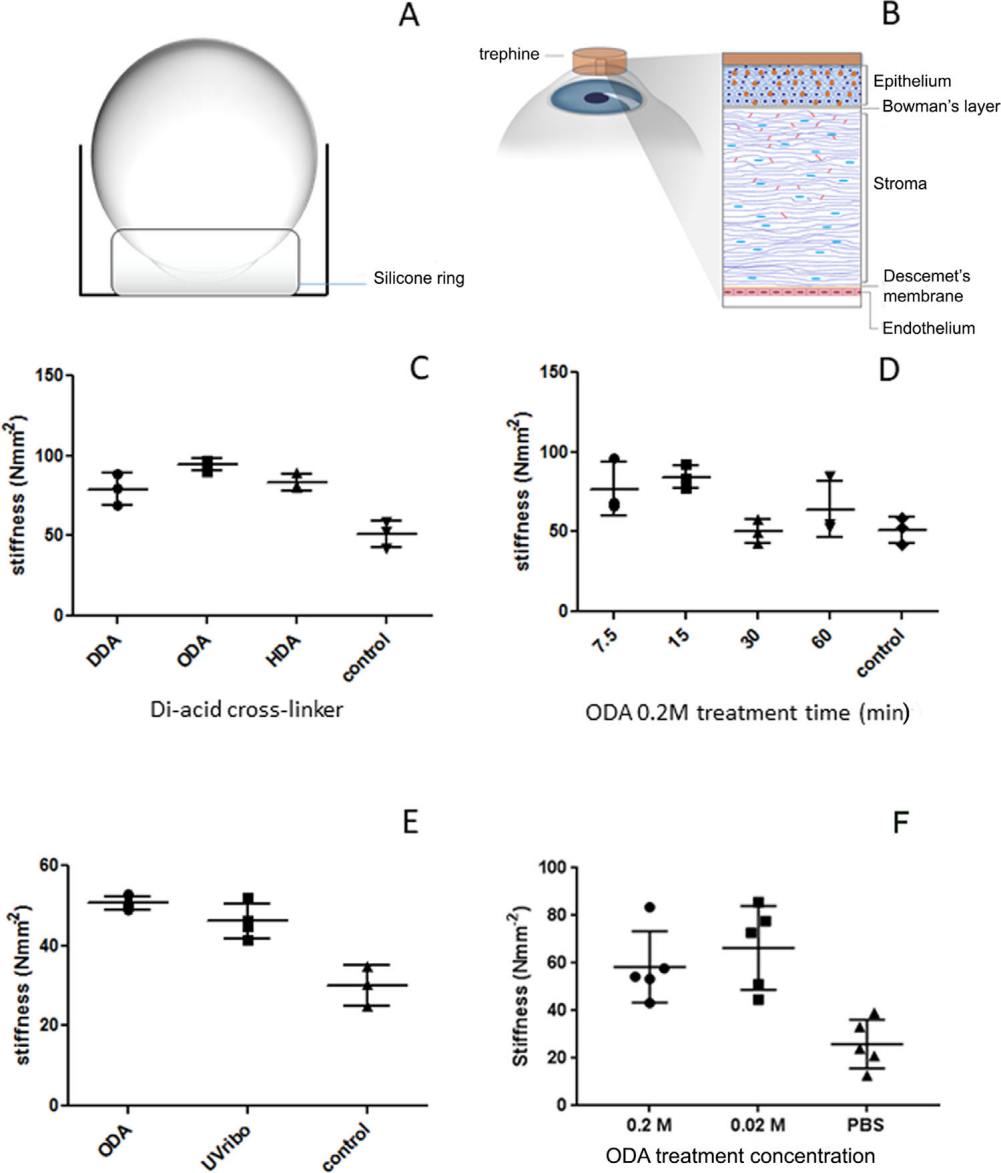
Analysis of mechanical stiffness of the porcine and human cornea after cross-linker treatment. (**A** and **B**) Diagrammatic representation of the treatment of porcine eyes and human eyes, respectively, with the chemical cross-linker. (**C**) Mechanical stiffness of ex vivo porcine cornea after treatment with dicarboxylic acids at 0.2 M concentration in comparison with PBS control. (**D**) Mechanical stiffness of ex vivo porcine cornea after treatment with 0.2 M cross-linker solution mixture containing ODA for various time periods. (**E**) Comparison of the mechanical stiffness of porcine cornea treated with 0.2 M cross-linker solution mixture for 15 minutes, UVA/riboflavin using the standard protocol and the PBS control. (**F**) Mechanical stiffness of human cornea treated with cross-linker solution mixture at 0.2 M and 0.02 M in comparison with PBS control.

Whole porcine globes were obtained from the abattoir (TanHouse Farm, Cheshire, UK) on the day of slaughter. Eyes were trimmed, cleaned, and stored in PBS (500 mL) that contained penicillin/streptomycin and amphotericin B (Gibco; Thermo Fisher Scientific, Waltham, MA, USA), overnight at 4°C. The next day whole eyes were placed into fresh cross-linking solution, cornea side down stabilized on silicone rings and incubated at 37°C for 15 minutes (Fig. 1A). After treatment the eyes were rinsed in PBS before analysis. We evaluated epithelial integrity of the porcine eyes immediately before and after treatment using fluorescein staining (Supplementary Fig. S1).

UVA/riboflavin cross-linking: The epithelial layer was scraped off using a scalpel blade and globes immersed cornea side down into 3 mL of 0.1% riboflavin solution (Aurolab, Madurai, India) for 30 minutes. The cornea was exposed to UV-A light (Opto Xlink Corneal Crosslinking System; Opto Electronica, São Paulo, Brazil; power 1.5 mW, intensity 3.003 mW/cm^2^) for 30 minutes while applying riboflavin solution (200 µL) to cornea every three minutes.

#### Human

Human donor cadaver eyes were received from Rotary Aravind International Eye Bank, Madurai, India. Normal human cadaver globes were harvested from the donor and processed within 24 to 30 hours after death. Keratoconus corneal central buttons (7 mm diameter) were obtained from patients who had severe keratoconus and were undergoing deep anterior lamellar keratoplasty procedure at the Aravind Eye Hospital, Madurai, India. Informed consent from the patients for the utilization of their corneas for research, institutional ethical committee approval of the Aravind Eye Care System were obtained (approval number: RES2015039CLI), and research followed the tenets of the Declaration of Helsinki. Human cadaver eyes were cleaned in PBS containing 2× penicillin and streptomycin solution (Life Technologies, Carlsbad, CA, USA), 50 µg/mL gentamicin (Life Technologies,), 1.25 µg/mL amphotericin B (Invitrogen, Carlsbad, CA, USA). The cadaver eyes were supported in a small dish with the epithelial surface facing up (Fig. 1B). A 10 mm trephine was placed over the corneal epithelial surface to cover the central and para-central epithelial surface and exclude the limbo-scleral region. The chemical cross-linker (0.2 M and 0.02 M) was applied within the trephine for 15 minutes at 37°C in a CO_2_ incubator. After the treatment, eyes were placed into a beaker of fresh PBS for washing. For all the experiments, PBS was used for untreated control.

It is important to note that the keratoconic tissue was treated differently to whole cadaver eyes. The keratoconus corneas were obtained from patients undergoing deep anterior lamellar keratoplasty procedures. They were treated with either concentration of the cross-linker only on the epithelial side by inverting the corneas in a 30 mm culture dish filled with 1 mL of the cross-linker solution. Care was taken to avoid the spilling or overflowing of the cross-linker solution into the stromal layer. After the 15 minutes treatment inside the 37°C incubator, the keratoconus corneas were washed with PBS, and the epithelium was scraped off using a sterile scalpel. The stroma was further processed in the same way as the stroma from the cadaver cornea.

### Mechanical Properties

Corneas were dissected from whole globes after treatment ensuring a perimeter of sclera remained and a dog-bone shape (10 mm long and 2 mm wide) sample cut using a custom-made punch. The width and thickness of the strip were measured using a digital calliper (Absolute Digimatic, Mitutoyo, UK) and then the stiffness of the porcine corneal strips was tested using a Linkam Tensile Tester with T95 stage (Linkam Scientific Instruments, Epsom, UK) with a 200 N load cell at a strain rate of 100 µm/s (n = 3 per group). The human corneal strips were tested (n = 5 per group) using an Instron tensile tester (Instron Corporation, Norwood, MA, USA) fitted with custom made tissue holding clamps and a 50 N load cell at a strain rate of 100 µm/s. Stiffness of the cornea were calculated from the linear part of the stress/strain graphs over a 5% strain region.

### Histology and Cell Culture

The tissue was prepared for histology using standard procedures, and the details can be found in the Supplementary Files. Explant cell cultures were prepared from the treated tissue as detailed in the supplementary Files.

### Penetration Assay (Porcine and Human)

Whole globes (porcine and human) were obtained and prepared for treatment as above. A 0.2 M concentration of NHS-fluorescein (ThermoFisher Scientific, St. Louis, MO, USA), was made up in PBS from a stock solution dissolved in dimethyl sulfoxide. The treatment solutions were applied to the prepared eyes for 15 minutes at 37°C in a CO_2_ incubator. After treatment, eyes were rinsed with PBS thoroughly before processing and embedding for histology.

### Animal Study

The animal study adhered to the ARVO Statement for the Use of Animals in Ophthalmic and Vision Research, and the NC3Rs (UK) and was performed by The Laboratory Animal Center, National Taiwan University College of Medicine (Institutional Animal Care and Use Committee Approval No. 20170110). Male, white New Zealand rabbits, 12 to 16 weeks old, were weighed before treatment and ketamine (40 mg/kg) anesthetic administered (n = 2 per condition and time point, total of six rabbits were used) and topical 0.5% proparacaine hydrochloride instilled into the eye (Bausch & Lomb, Rochester, NY, USA). An eyelid speculum was placed in the left eye to keep it open for treatment; the right eye served as a control. In the left eye the lack of corneal staining with fluorescein was used to determine the integrity of the corneal epithelium (fluorescein sodium 1% w/v; Bausch & Lomb) and photographed. The fluorescein was washed away with saline solution. The rabbit was orientated to hold the treatment solution (0.2 M) in place for 15 minutes, after which the solution was drained, and the eye was washed with copious amounts of saline solution. The integrity of the epithelium was again checked using 1% fluorescein and photographed, the eyes were rinsed with saline solution, antibiotic drops were administered in the treated eyes, and the rabbits were allowed to recover.

The rabbits were assessed on alternate days to observe any changes in animal behavior, determine any adverse ocular findings resulting from chemical cross-linking, and perform corneal photography. At the relevant time points (one, three, and seven days after treatment) rabbits were sedated, an eyelid speculum was placed to keep eyes open, and the corneal epithelium integrity was evaluated by fluorescein application and photographed using a mounted microscope (Carl Zeiss, Inc., White Plains, NY, USA; ExwaveHAD; DSP 3CCD, color video camera, Sony, Tokyo, Japan). The rabbits were euthanized using an overdose of intravenous pentobarbital (100 mg/kg), and the eyes were enucleated. The tissue was fixed in 4% paraformaldehyde and processed for histological analysis in hematoxylin and eosin (H&E) and TUNEL staining. Epithelium thickness was measured using ImageJ (NIH); three sections were taken from each tissue per each time point to obtain six measurements. Further details of methods can be found in the Supplementary Materials.

### Results

#### Chemical Cross-Linking of Tissue to Increase Stiffness

We demonstrated an increase in the stiffness of ex vivo porcine and human corneal tissue using our dicarboxylic acid ester activated by a carbodi-imide chemical reaction. Initially cross-linking solutions containing one of three dicarboxylic acids, DDA, ODA, and HDA at a 0.2 M concentration were evaluated using ex vivo porcine eyes. Results show a significant increase (*P* < 0.05) in the stiffness of 54.8% (DDA), 84.9% (ODA), and 63.6% (HDA) in comparison to the untreated control tissue (Fig. 1C). Of the three dicarboxylic acids investigated ODA exhibited the highest increase in stiffness, and, hence, cross-linker solution mixture containing ODA was used for the remaining experiments. In these experiments the epithelium was not removed, but it is important to consider that although the porcine eyes were tested as soon as possible after death, the epithelial cell tight junctions may have been compromised to some extent postmortem.

We evaluated the influence of treatment times of the ODA containing cross-linker mixture from 7.5 to 60 minutes and demonstrated there was no statistically significant difference over this range (Fig. 1D). The highest increase in stiffness (84.5%), considered to be clinically relevant, was obtained after 15 minutes exposure and was therefore used as the treatment time for all further experiments. Using our ex vivo porcine model we compared the cross-linker with the conventional clinical cross-linking method and demonstrated that similar increases in stiffness were achieved between the cross-linker solution mixture (0.2 M) treatment (50.6%) and UVA/riboflavin treatment (46.1%), both of which were significantly different (*P* < 0.05) than untreated control tissue (Fig. 1E). In these tests the epithelium was removed for the UVA/riboflavin treatment but left intact for the chemical cross-linking treatment. Whole human globes were treated with the cross-linking solution at concentrations of 0.2 M and 0.02 M. We demonstrated a significant increase in stiffness; 125.5%, (*P* < 0.05) after treatment with 0.2 M cross-linker and 156.3% (*P* < 0.05) after treatment with 0.02 M solution compared to untreated control tissue (Fig. 1F). The difference between 0.2 M and 0.02 M treated tissues was not statistically significant. We used NHS-Fluorescein to assess penetration of the cross-linker into the tissue. 0.2 M NHS-Fluorescein crossed the corneal epithelial barrier, penetrated deep into the stroma but did not reach the endothelium (Supplementary Fig. S2).

#### Safety Evaluation of the Chemical Cross-Linker

**Figure 2.**
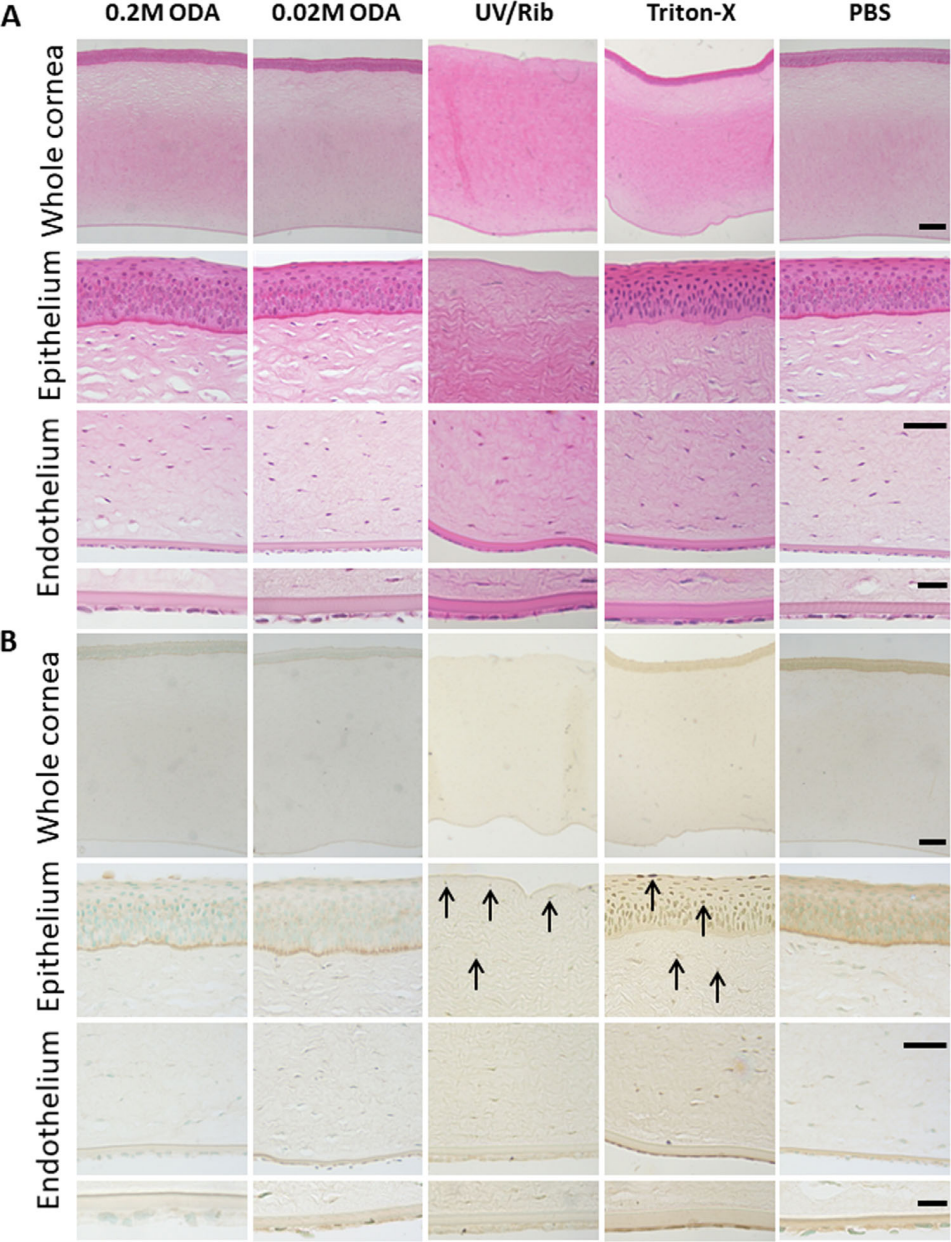
Effect of the cross-linker on the gross morphology and cytotoxicity in the porcine cornea. (**A**) Representative micrographs of H&E staining of porcine corneal tissue after cross-linking with the cross-linker solution mix (indicated as ODA) showing intact epithelium and endothelium. UVA/riboflavin treated cornea has the epithelium removed and shows few keratocytes in the anterior stroma (*scale bars*: 100, 20, and 10 µm, respectively). (**B**) In situ apoptosis detection TUNEL assay demonstrating absence of any signs of apoptosis in treated cornea, Triton-X treated tissue demonstrates apoptotic cells throughout the epithelium and endothelium (*scale bars*: 100, 20, and 10 µm, respectively). *Arrows* indicate the apoptotic cells as shown by the *dark brown staining* in the nuclei of cells.

**Figure 3.**
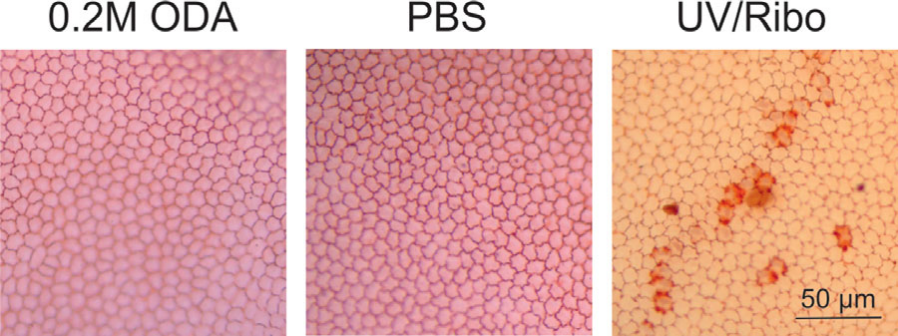
Effect of the cross-linker on porcine endothelium. Representative micrographs of alizarin red– and trypan blue–stained porcine endothelium demonstrating an intact monolayer of endothelial cells in the cross-linker–treated (indicated as ODA) and PBS-treated cornea with evidence of mild damage to the endothelial cells after treatment with UVA/riboflavin. *Scale bar*: 50 µm.

**Figure 4.**
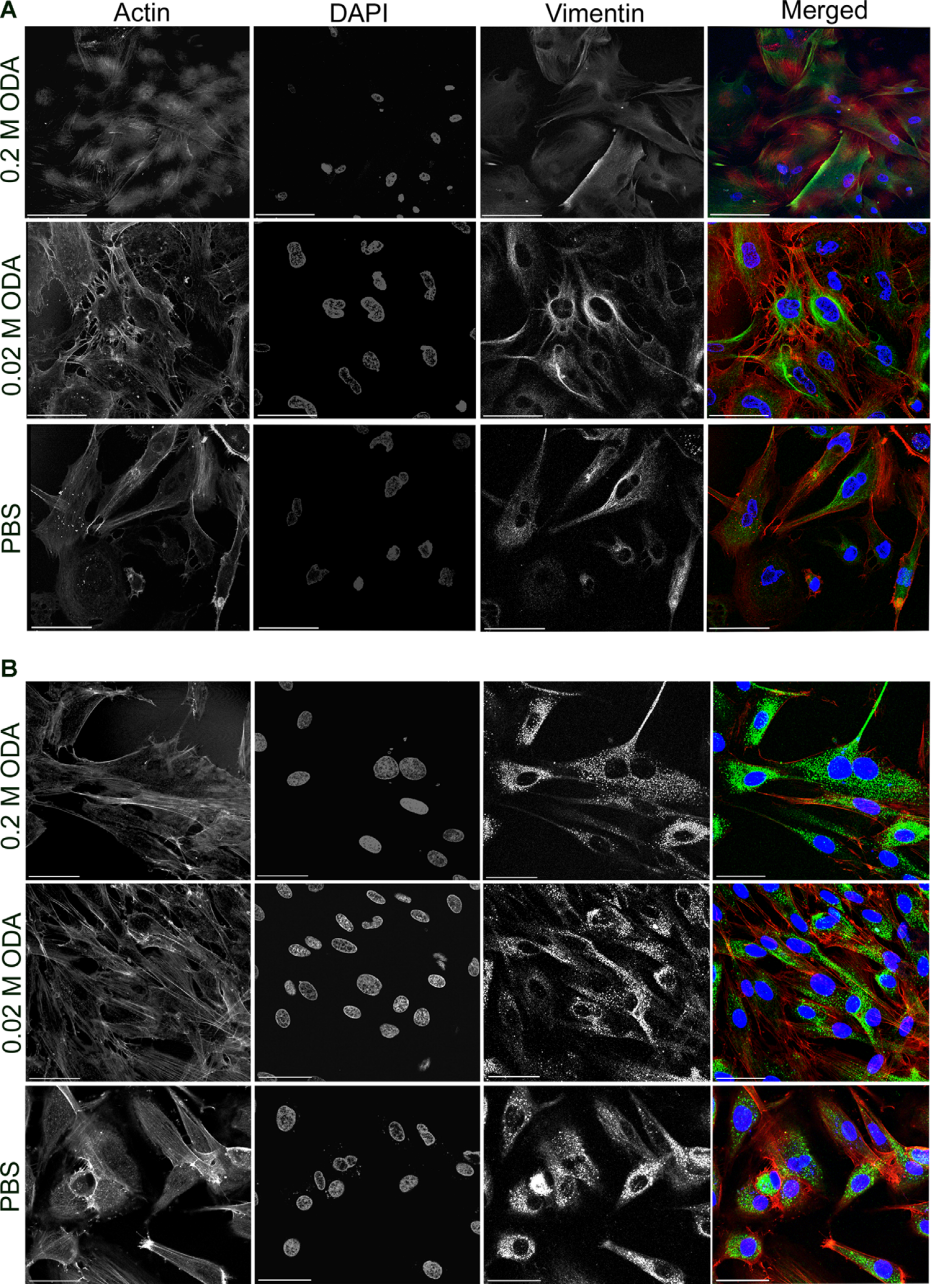
Representative micrographs of stromal fibroblasts cultured from human cadaver (**A**) and keratoconic (**B**) corneal stromal tissue after treatment with 0.2 M and 0.02 M cross-linker solution mixture (labeled as ODA) in comparison with PBS control demonstrating survival of the cells. Expression of vimentin (*green*), the stromal fibroblast specific marker and actin network (*red*) is seen unperturbed even after treatment with different concentrations of the cross-linker. *Scale bars*: 102 µm for 0.2 M images and 43.5 µm for 0.02 M and PBS images.

We analyzed the effect of the cross-linker on the gross morphology of the cornea and its cytotoxicity on cells of the corneal layers. We demonstrated that histological analysis of porcine cornea treated with 0.2 M chemical cross-linker had the same gross morphology as the untreated cornea. The epithelium was intact, and there was no evidence of death of the stromal keratocytes (Fig. 2A). In comparison, the UVA/riboflavin treated cornea showed evidence of a lack of stromal cells in the uppermost section of the tissue, suggesting the treatment had killed the cells. Apoptosis of the cells was evaluated using TUNEL staining and demonstrated no evidence of apoptosis in the cross-linker treated tissue or the control whereas Triton-X treated samples demonstrated apoptotic cells (Fig. 2B) as evidenced by the dark brown staining in the nuclei of the cells in the epithelium and stroma. Similar results were obtained for treated and untreated human cadaver cornea (Supplementary Fig. S3). Alizarin red/trypan blue staining was used to evaluate potential damage to the endothelium, which identifies the cell membrane in intact cells and the permeability of damaged cells,^16^ and in all samples a monolayer of intact endothelial cells was observed (Fig. 3) over the majority of the endothelium. To confirm the survival of corneal cells after chemical cross-linker treatment further, we harvested explants from treated corneal tissue and allowed cells to grow out in culture using standard procedures. Stromal cells were observed to grow out from all tissue explants (porcine; Supplementary Fig. S4, normal human; Fig. 4A and keratoconic human; Fig. 4B) after treatment. Human culture expressed the stromal cell specific marker vimentin. This is additional evidence to suggest that the cross-linker has a lower cytotoxic effect to the stromal cells in comparison with UVA treatment.

#### In Vivo Safety Analysis of the Cross-Linker

**Figure 5.**
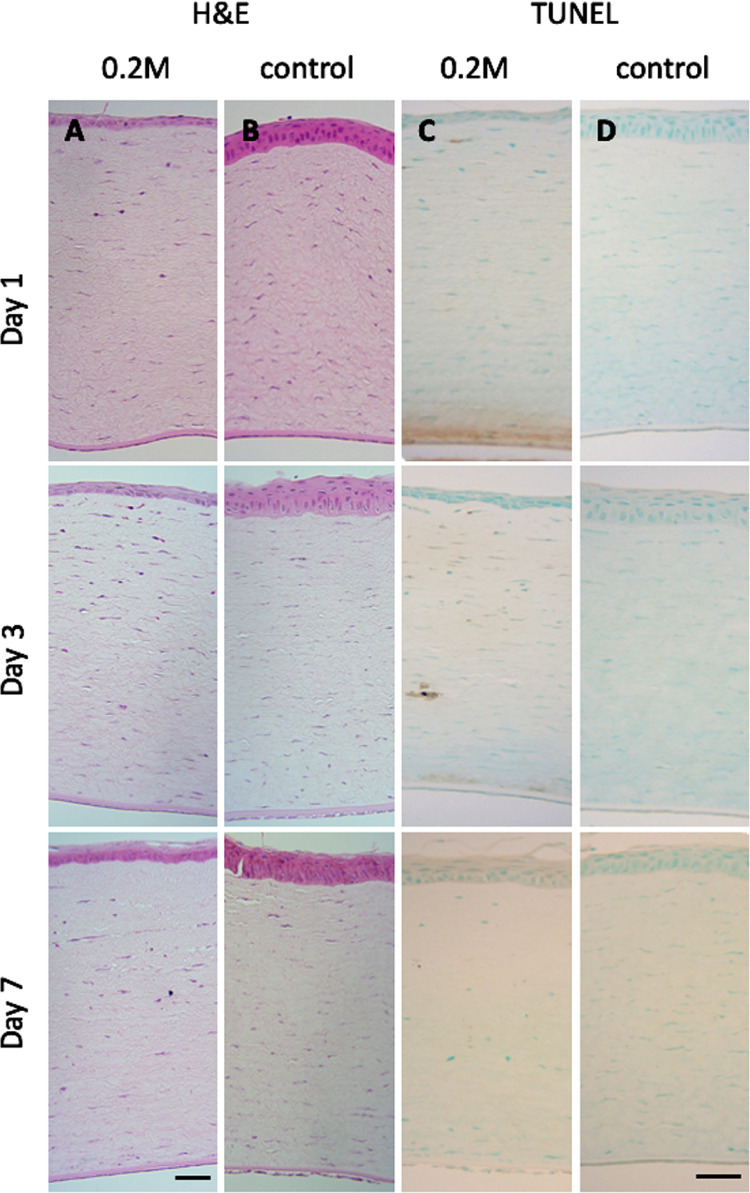
Effect of the cross-linker on the gross morphology and cytotoxicity of rabbit cornea. Representative micrographs of H&E stained samples (**A** and **B**) and TUNEL assay (**C** and **D**) of rabbit cornea, taken at days 1, 3, and 7 after treatment with 0.2 M cross-linker solution and the PBS controls. H&E shows thinning of corneal epithelium; however, TUNEL assay is negative for apoptosis. *Scale bar*: 20 µm.

In vivo safety of the cross-linker treatment was studied in a short-term rabbit model. At 24 hours after treatment it was clear that the rabbits had some discomfort (Supplementary Fig. S5), and at this stage there was thinning of the epithelium as assessed qualitatively (Fig. 5). By three days the rabbits appeared unaffected by the treatment and histologically the epithelium had recovered by day seven (Supplementary Table S1), although the regularity of the cells was abnormal. It was clear the thickness of the epithelium and eyelid had been impacted by the treatment. Histologically there was no evidence of cell loss in the stroma at any time point, and the endothelium was intact throughout. TUNEL staining did not show any evidence of apoptosis (Fig. 5).

## Discussion

In this study, we have developed a novel chemical cross-linker that can be applied topically to the cornea without removal of epithelium resulting in stiffening of the cornea. We report here for the first time the potential of a dicarboxylic acid cross-linker activated by NHS/EDCI to produce clinically relevant stiffening of the cornea as a potential clinical treatment. In conventional corneal cross-linking, the corneal epithelium is removed to allow riboflavin to penetrate into the stroma, then the cornea is exposed to UVA irradiation. Riboflavin is a strong absorber of UVA, specifically at the wavelengths used, and it is this increased absorption of UVA that causes cytotoxicity to the corneal cells.^17^ Damage to keratocytes causes a pseudo-haze in the cornea due to edema in the keratocyte lacunae for three to four months after treatment.^2^ A very important concern about the use of UVA is its toxicity to the corneal endothelium. This means that conventional treatment cannot be used in keratoconus patients with thin corneas and given that keratoconus is a corneal thinning disorder, and many patients are identified at the later stages of the condition, particularly in the developing world, withholding conventional treatment to halt disease progression on the basis of corneal thickness is a significant clinical problem, and these patients are likely to go on to require corneal transplantation.

The importance of our study is that the cross-linking treatment stiffens the ex vivo cornea to the same extent as the UVA-riboflavin conventional treatment with a treatment time of 15 minutes and that it does not require removal of the epithelium and lower toxicity to corneal cells. We believe this would make the treatment applicable to a greater proportion of patients, particularly those with more advanced disease and thus thinner cornea. As the cross-linker is applied to the cornea, it will begin to interact with the proteins as it diffuses into the tissue. As cross-links form, they will stiffen the tissue, which could also reduce the rate of diffusion further into the stroma. In this way the likelihood of the cross-linker reaching the corneal endothelium is reduced, thus protecting it from damage. We used a fluorescently-tagged NHS and demonstrated its penetration deep into the corneal tissue through the intact epithelium (Supplementary Fig. S2). Although the fluorescein-NHS is not the same as the cross-linker they both have NHS-ester linkages that will interact with primary amines in the tissue and so provide a realistic representation of the likely penetration of the cross-linker that can be visualized.

Similar to previously published literature,^2^^,^^17^ the UVA/riboflavin-treated porcine cornea had evidence of a lack of stromal cells in the uppermost section of the tissue, suggesting the treatment had killed the cells. In our treatments, keratocyte cell death was not observed either by H&E or TUNEL staining in ex vivo porcine and human corneas, as well as in the in vivo rabbit corneas. Furthermore, outgrowth of stromal cells from treated explants, specifically human and keratoconic tissue, confirms the survival of the keratocytes after the chemical cross-linking treatment.

The cross-linker solution has an acidic pH during the course of treatment of the corneas (Supplementary Fig. S6), and we believe it is the low pH that caused discomfort for the rabbits, swelling of the eye lids and thinning of the corneal epithelium; however, at no point was a full thickness epithelial defect observed. Other eye drops, for example for glaucoma treatment, have a pH in the 4-7.4 range.^18^ There is, however, an opportunity to raise the pH of the cross-linker closer to seven and still cause cross-linking within the tissue, to reduce discomfort in in vivo conditions. EDCI is known to be most effective at forming esters at pH 4.5 to 7.2^19^^,^^20^ and NHS is similarly known to function most effectively between pH 7.5 to 8.5.^21^ Therefore at pH 7 both NHS and EDCI should still be able to react with ODA and promote cross-linking. We also intend to modify the treatment procedure in the future using a dedicated corneal suction ring device to limit the treatment solution to the central cornea only and so protect the surrounding tissues. This would also provide greater protection of the limbal epithelial cells, which could increase the rate of epithelial healing should thinning continue to occur.

Application of this cross-linker does not require specialized equipment, such as that needed to deliver UVA/riboflavin and thus in the longer term could be used to treat patients within their local community healthcare centers, rather than requiring transport to hospital ophthalmology clinics. This could have a significant bearing of the treatment of this sight-threatening condition globally in low resource settings. Demonstration of the ability to stiffen tissue in this way could also have potential application in other ocular conditions, for example, other reasons for loss of corneal shape, inflammation in the eye caused by infective, traumatic or immune-mediated corneal or scleral disorders, and mechanical strengthening of a weakened sclera due to myopia^22^ or glaucoma.^23^ There are published trials of using conventional cross-linking protocol as an adjuvant therapy for treating fungal keratitis along with the usual antifungal drugs,^24^^,^^25^ bullous keratopathy, and other corneal ectasias.^10^ Our novel chemical cross-linker would be a promising potential alternative to be used as an adjuvant therapy for these disease conditions where survival of corneal stromal cells would be a significant clinical advantage. Other corneal cross-linking agents like 0.25% genipin^26^ or sodium hydroxymethylglycinate^27^ had a comparable cross-linking efficiency to the conventional treatment with minimal toxicity to endothelial cells in ex vivo porcine and rabbit models. In all these trials, the cross-linker was applied after epithelial debridement. The conventional cross-linking protocol and its modified accelerated protocols also formed a demarcation line in the posterior cornea which was indicative of keratocyte apoptosis observed in cross-linked human corneas.^8^ A couple of cross-linkers were designed to be applied without the removal of epithelium. Short chain aliphatic beta nitro-alcohols exhibited less cytotoxicity and cross-linked porcine corneal strips to a great extent as measured by thermal shrinkage temperature compared with the standard treatment.^28^ Wang et al.^29^ used EDCI, NHS and chondroitin sulphate based cross-linker in an ex vivo rabbit model of keratoconus. The cross-linker could enhance the stiffness of the cornea, restore collagen alignment without being cytotoxic to keratocytes and the epithelium. We did not observe keratocyte apoptosis in the stroma or a demarcation line in the stroma after treatment with our chemical cross-linker, and we observed no change in the endothelial layer in porcine, rabbit, and human corneas. It was clear, however, that the thickness of the rabbit epithelium and eyelid had been impacted by the treatment. Further work is needed to adjust the pH of the formulation to closer to pH 7 and in the future we intend to modify the procedure to use a dedicated ring device to limit the treatment solution to the central cornea alone to protect the surrounding tissues.

In conclusion, this study demonstrates for the first time the potential of a dicarboxylic acid cross-linker activated by NHS/EDCI to produce clinically relevant stiffening of the cornea for treatment of keratoconus. We show that the cross-linker can penetrate into the corneal stroma without removal of the epithelium, which will be a significant advantage for patient care. We also demonstrate that it caused no histological changes to ex vivo tissue and an initial in vivo study demonstrated survival of the corneal cells in a rabbit model. This treatment has several advantages over the conventional UVA/riboflavin treatment in terms of there being no need to physically remove the epithelium and a lower toxicity throughout the cornea.

## Supplementary Material

Supplement 1

Supplement 2

Supplement 3

Supplement 4

Supplement 5

Supplement 6

Supplement 7

Supplement 8
