# Supplementary material for: Chemical Cross-Linking of Corneal Tissue to Reduce Progression of Loss of Sight in Patients With Keratoconus
Source: Transl Vis Sci Technol. 2021 Apr 29;10(5):6. doi: 10.1167/tvst.10.5.6 (PMC8088226; doi:10.1167/tvst.10.5.6)
Supplement: Supplement 1 [file tvst-10-5-6_s001.pdf]

Pre-crosslinking treatment (after PBS overnight)

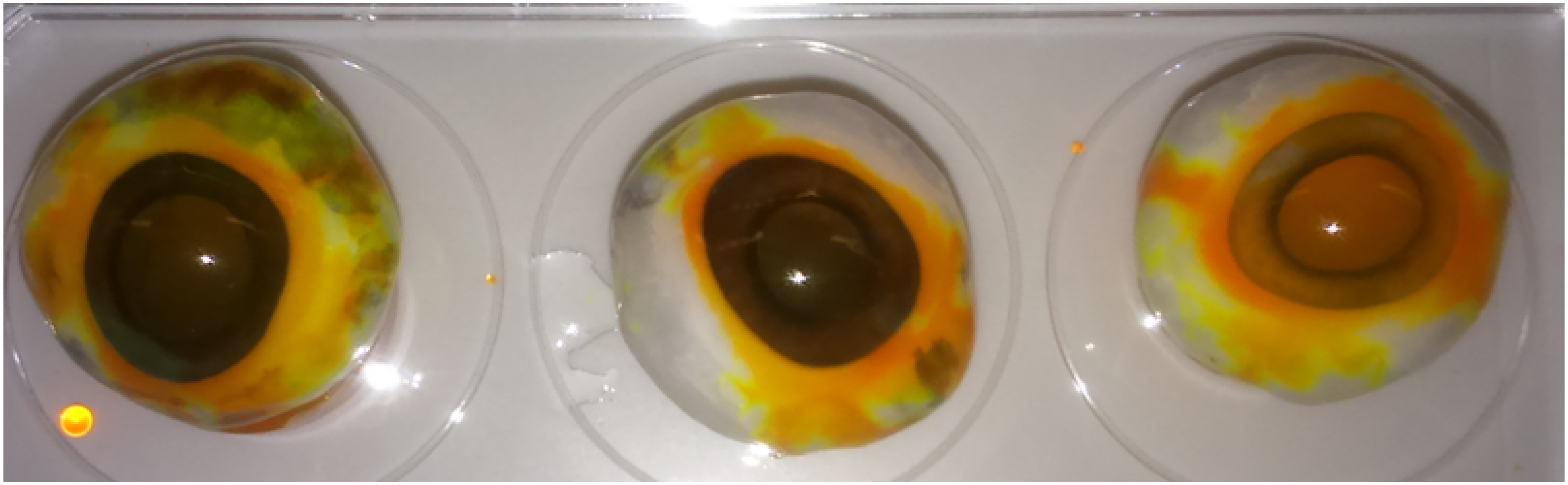

Post-crosslinking treatment

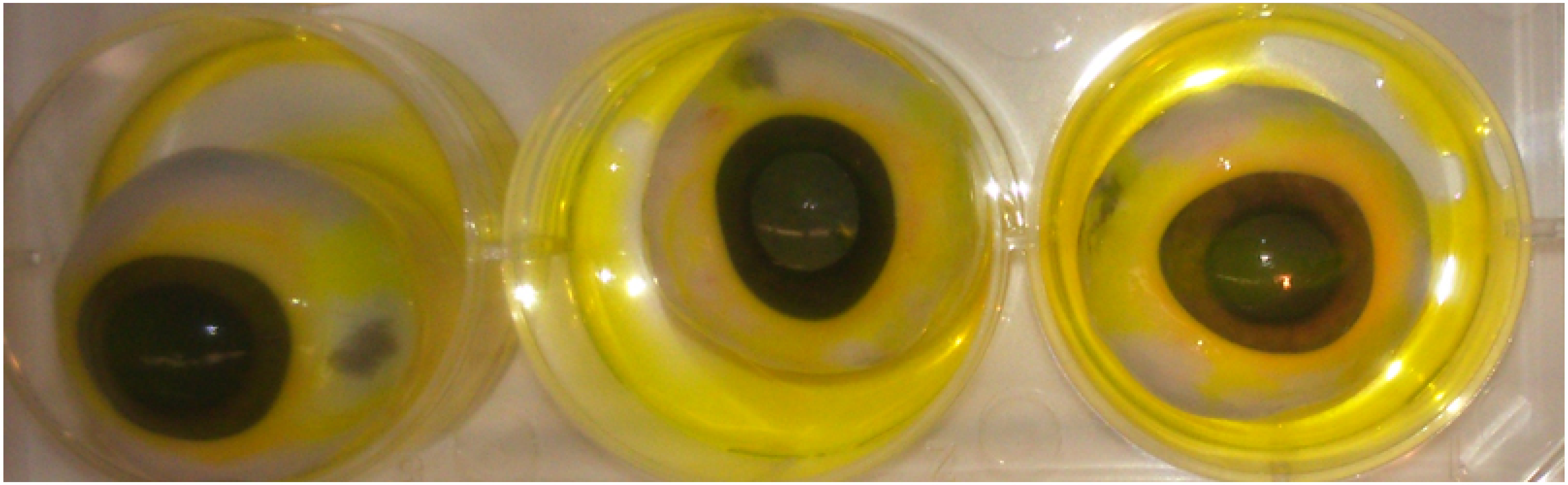

Supplementary figure S1: Representative photograph of porcine globes treated with fluorescein to confirm epithelium integrity, after storage in PBS overnight and before and after cross-linker treatment.
