# Supplementary material for: Chemical Cross-Linking of Corneal Tissue to Reduce Progression of Loss of Sight in Patients With Keratoconus
Source: Transl Vis Sci Technol. 2021 Apr 29;10(5):6. doi: 10.1167/tvst.10.5.6 (PMC8088226; doi:10.1167/tvst.10.5.6)
Supplement: Supplement 2 [file tvst-10-5-6_s002.pdf]

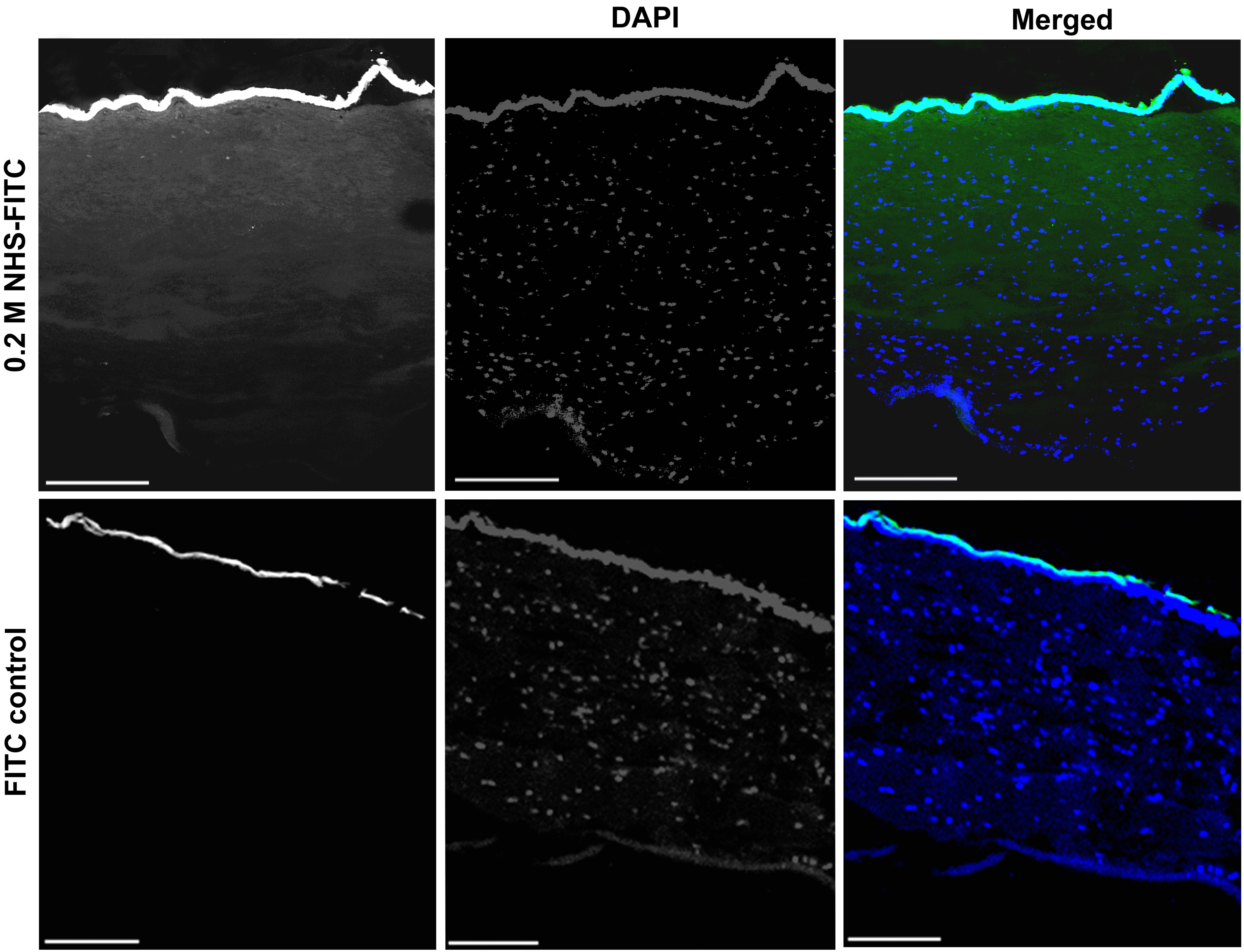

Supplementary figure S2: Penetration of the cross-linker into the cornea without removing the epithelium. Representative micrographs demonstrating the penetration of FITC labelled NHS into the human cadaver stroma without removing the epithelium. Scale bar = 200 μm.
