# Supplementary material for: Chemical Cross-Linking of Corneal Tissue to Reduce Progression of Loss of Sight in Patients With Keratoconus
Source: Transl Vis Sci Technol. 2021 Apr 29;10(5):6. doi: 10.1167/tvst.10.5.6 (PMC8088226; doi:10.1167/tvst.10.5.6)
Supplement: Supplement 3 [file tvst-10-5-6_s003.pdf]

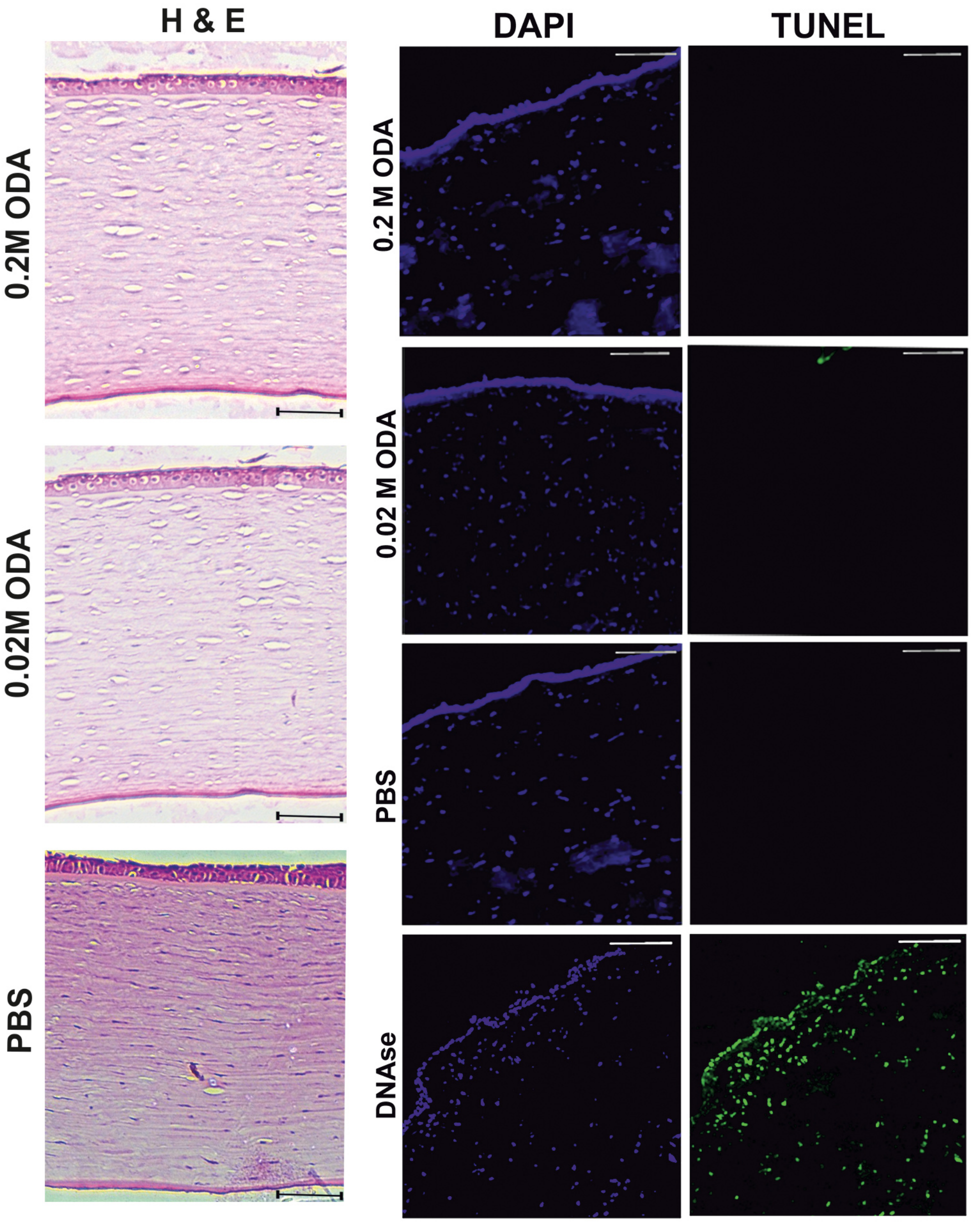

Supplementary figure S3: Effect of the cross-linker on the gross morphology and cytotoxicity of human cadaver cornea. Representative micrographs of human cornea after treatment with 0.2 M and 0.02 M cross-linker solution mixture (represented as ODA) in comparison with PBS control stained with H&E and a fluorescent TUNEL assay demonstrating maintenance of the gross ultrastructure of the tissue and no evidence of apoptosis. DNAse treated corneal tissue is used as a positive control for TUNEL signal. The fluorescent signal represents the fragmented DNA molecules inside the nuclei. Scale bar is 100 μm.
