# Supplementary material for: Chemical Cross-Linking of Corneal Tissue to Reduce Progression of Loss of Sight in Patients With Keratoconus
Source: Transl Vis Sci Technol. 2021 Apr 29;10(5):6. doi: 10.1167/tvst.10.5.6 (PMC8088226; doi:10.1167/tvst.10.5.6)
Supplement: Supplement 4 [file tvst-10-5-6_s004.pdf]

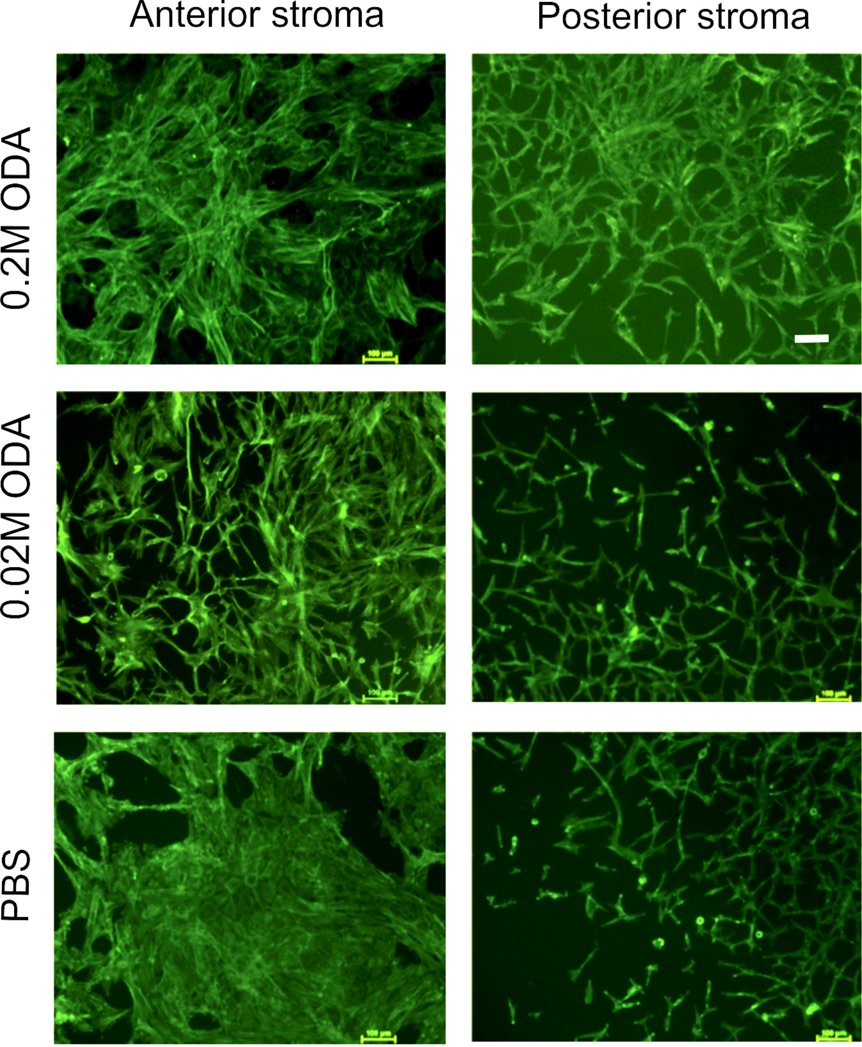

Supplementary figure S4: Representative micrographs of corneal stromal fibroblasts from the anterior and posterior sections of porcine cornea after treatment with 0.2 M, 0.02 M cross-linker solution mixture (indicated as ODA) and PBS control demonstrating a healthy culture of stromal cells from all tissue explants. Scale bar is 100 μm.
