# Supplementary material for: Chemical Cross-Linking of Corneal Tissue to Reduce Progression of Loss of Sight in Patients With Keratoconus
Source: Transl Vis Sci Technol. 2021 Apr 29;10(5):6. doi: 10.1167/tvst.10.5.6 (PMC8088226; doi:10.1167/tvst.10.5.6)
Supplement: Supplement 5 [file tvst-10-5-6_s005.pdf]

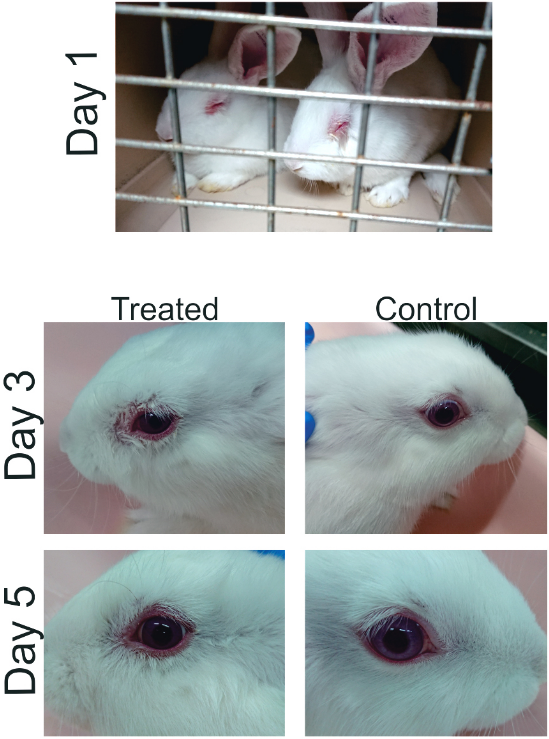

Supplementary figure S5: Gross evaluation of rabbit eyes after treatment. Representative photographs of the rabbits treated with 0.2 M cross-linker solution mixture and untreated eyes, at day 1 post treatment (top) demonstrating some reduced eye opening and periocular inflammation and at day 3 (middle) and day 5 (bottom). The reduced eye opening and periocular inflammation were resolved by day 3. Future work intends to assess the impact when using a ring to contain the treatment solution on the cornea only.
