# Supplementary material for: Chemical Cross-Linking of Corneal Tissue to Reduce Progression of Loss of Sight in Patients With Keratoconus
Source: Transl Vis Sci Technol. 2021 Apr 29;10(5):6. doi: 10.1167/tvst.10.5.6 (PMC8088226; doi:10.1167/tvst.10.5.6)
Supplement: Supplement 6 [file tvst-10-5-6_s006.pdf]

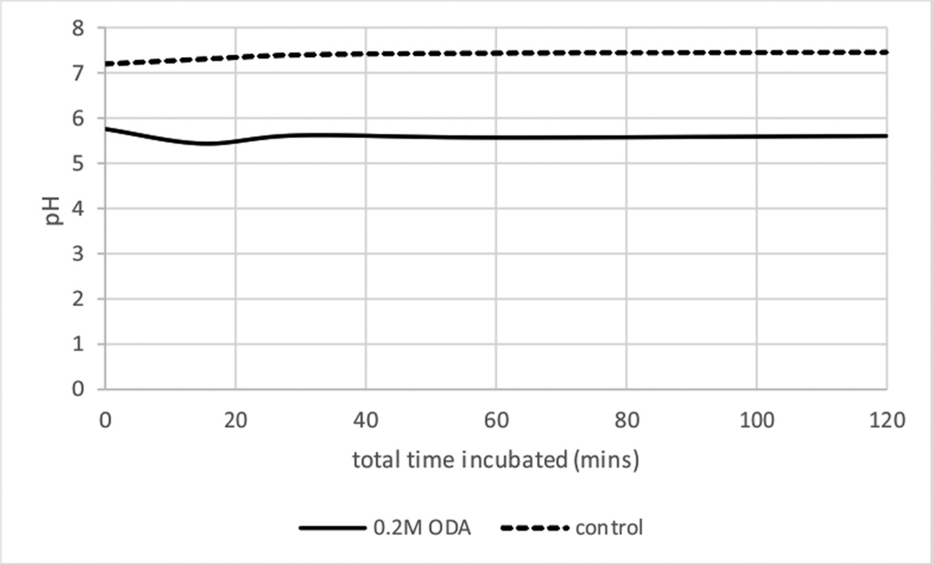

Supplementary figure S6: Graph showing pH change of crosslinker solution over time. The crosslinker solution is slightly acidic.
