# Supplementary material for: Chemical Cross-Linking of Corneal Tissue to Reduce Progression of Loss of Sight in Patients With Keratoconus
Source: Transl Vis Sci Technol. 2021 Apr 29;10(5):6. doi: 10.1167/tvst.10.5.6 (PMC8088226; doi:10.1167/tvst.10.5.6)
Supplement: Supplement 7 [file tvst-10-5-6_s007.pdf]

Supplementary material:

Supplementary methods

Histological analysis:

Porcine corneas were fixed in 4% neutral buffered formalin (NBF) for 20 minutes at room temperature (RT), processed (Leica TP 1020, Leica BiosystemsNussloch GmbH, Germany) and wax embedded (Leica EG 1150c, Leica BiosystemsNussloch GmbH, Germany). Sections (7 µm) were cut using a microtome (Leica RM2235, Leica Biosystems Nussloch GmbH, Germany) and stained with haematoxylin and eosin using standard laboratory protocols. Sections were stained for TUNEL using the protocol provided in an *in situ* apoptosis detection kit (Abcam, UK). All sections were imaged using an optical microscope (OLYMPUS BX60, Olympus Optical Co Ltd., Japan)

To stain the endothelium, corneas were excised from the whole globe ensuring a perimeter of sclera was allowed to remain and washed gently by dipping into a beaker of PBS. They were carefully placed on a surface with the endothelium facing upwards. 2.5% (w/v) trypan blue solution (500 µl) was dropped onto the endothelium and incubated for 30 seconds at RT. The cornea was washed by dipping into a beaker of PBS, followed by dropping 1% (w/v in PBS) alizarin red solution (BDH laboratory reagents) onto the endothelium and incubated for 2 minutes at RT and then washed by dipping into a beaker of PBS.[16] Corneas were blotted carefully on tissue, without touching the endothelium, and placed on a glass slide epithelium side down. Stained endothelium were imaged using an optical microscope (OLYMPUS BX60).

TUNEL staining in human corneas:

Human cadaver corneas dissected from cadaver globes after the pseudo-clinical treatment of the cross-linker solution and the keratoconus corneas obtained from patients undergoing deep anterior lamellar keratoplasty (DALK) corneal transplantation procedure at Aravind Eye Hospital (Madurai, India) were used for TUNEL staining. For the keratoconus corneas, the

cross-linker solution was applied only to the epithelial side. The data from human cadaver corneas alone is represented here. 8 µm cryosections of the dissected corneas were taken in a cryo-microtome (Leica, Germany). Sections were fixed with 4% paraformaldehyde and then blocked with 3% H_2O_2 (Merck) for 15minutes. The sections were permeabilized with sodium citrate buffer (0.1% sodium citrate and 0.1% Triton x- 100 in PBS (Fluka) for 2 minutes on ice. After a wash with PBS, TUNEL mixture (Roche diagnostics, Switzerland) was added to the sections and incubated at 37°C for 1 hour in a humidified chamber. The sections were then washed and mounted in Vectashield® Antifade Mounting Medium (Vector labs, USA) containing 5 µg/ml DAPI. Images were taken using a 10X objective of a confocal laser scanning microscope (Leica, TCS SP8, Germany).

pH changes

The pH of solutions of ODA, NHS and EDCI were measured with a calibrated pH meter (Hanna Instruments, USA) immediately after mixing (giving a 0.2 M solution) and thereafter every 15 minutes incubation (at 37°C), up to 120 minutes. PBS was used as a control. Each solution was agitated before the pH was taken and before placing back into the incubator.

Cell culture method:

After treatment with the cross-linking solutions or PBS, the porcine eyes were washed with sterile PBS and the corneas dissected and the ciliary body removed under laminar flow. The epithelium and endothelium were removed by scraping with a scalpel, the sclera rim was trimmed away and the stroma washed with sterile PBS. The stroma was cut in half to dissect the anterior side of the cornea from the posterior side, and then cut into small pieces using a scalpel. The tissue pieces were placed in a 24 well plate (1 eye per well), a small drop of complete media placed on top of the explant. The plates were placed into the incubator (37°C, 5% CO_2) to allow the explants to stick to the bottom of the plate. Once the tissue pieces had stuck down they were covered with media (DMEM/F12 HAM, 10% FCS,

1% penicillin/streptomycin/Amphotericin B) (1000 µl) and incubated at 37°C, 5% CO_2. Media was changed every two days by removing 500 µl and replacing with 500 µl fresh media. The tissue samples were cultured for 17 days. Media was removed from the wells and washed with PBS (3 x 1 ml). The cells were fixed with 10% NBF at room temperature for 10 minutes and then stained with phalloidin (Thermofisher Scientific, USA) and DAPI using the standard procedure and imaged using a fluorescence microscope (Nikon ECLIPSE Ti, Japan).

For the human eyes the stroma from the cross-linker treated cadaver cornea was cut into pieces and digested with 3.3 mg/ml collagenase (Sigma) for 8 h at 37°C in a CO_2 incubator followed by 0.05% trypsin- EDTA (Invitrogen) treatment for 15 minutes. The separated cells of the stroma were spun and the pellet was resuspended in DMEM-F12 medium containing 10% foetal bovine serum (Invitrogen, USA) and seeded on coverslips. The cells were allowed to attach and spread on the coverslips. For immunostaining, cells were fixed with 4% paraformaldehyde (PFA), and immunostained with Vimentin antibody (Cell Signaling,USA) using the standard procedure. Then phalloidin-TRITC was added to the cells at 1:40 dilution and incubated at 1 hour at RT. The cells were mounted with DAPI containing mounting medium and imaged in a confocal laser scanning microscope (Leica-TCS SP8, Germany).

Statistical analysis for the rabbit experiment:

The triplicate values of two eyes from each condition (N=6) were used to derive the interquartile range and median at each time-point for the 0.2 M cross-linker treated group and the control group. Mann Whitney U test (non-parametric) was used for the analysis of significance between the control and treated groups. P value ≤ 0.05 is considered as statistically significant. The statistical analysis was done using STATA ver. 14 software (Texas, USA).
