# Supplementary material for: Chemical Cross-Linking of Corneal Tissue to Reduce Progression of Loss of Sight in Patients With Keratoconus
Source: Transl Vis Sci Technol. 2021 Apr 29;10(5):6. doi: 10.1167/tvst.10.5.6 (PMC8088226; doi:10.1167/tvst.10.5.6)
Supplement: Supplement 8 [file tvst-10-5-6_s008.pdf]

Supplementary table S1: Table exhibiting thickness of rabbit epithelium after cross-linker treatment

Duration	0.2 M group Median (IQR) (μm)	Control group Median (IQR) (μm)	P value *
Day 1	4.3 (3.01 – 5.16)	12.04 (11.61 – 12.47)	0.0038 (S)
Day 3	4.94 (4.3 – 5.59)	14.83 (13.76 – 15.48)	0.0038 (S)
Day 7	9.89 (8.17 – 12.04)	11.39 (9.46 – 13.76)	0.1954 (NS)

N=6, from triplicate readings of two eyes in each condition; IQR – Interquartile Range;
*-Mann Whitney U test; S – Significant ($p \leq 0.05$); NS – Not Significant

Measurements were taken using histology images of the cornea in Figure 5. Measurements show initial thinning and then some recovery of the epithelium with time in 0.2 M crosslinker treated group.
